# Review of Hereditary and Acquired Rare Choreas

**DOI:** 10.5334/tohm.548

**Published:** 2020-08-06

**Authors:** Daniel Martinez-Ramirez, Ruth H. Walker, Mayela Rodríguez-Violante, Emilia M. Gatto, on behalf of the Rare Movement Disorders Study Group of International Parkinson’s Disease

**Affiliations:** 1 Tecnologico de Monterrey, Escuela de Medicina y Ciencias de la Salud, Monterrey, MX; 2 Departments of Neurology, James J. Peters Veterans Affairs Medical Center, Bronx, NY, US; 3 Mount Sinai School of Medicine, New York, NY, US; 4 Movement Disorders Clinic, National Institute of Neurology and Neurosurgery, Mexico City, MX; 5 Department of Neurology, Affiliated University of Buenos Aires, Buenos Aires, AR

**Keywords:** Rare disease, orphan disease, inherited disease, treatment

## Abstract

**Background:**

Movement disorders are often a prominent part of the phenotype of many neurologic rare diseases. In order to promote awareness and diagnosis of these rare diseases, the International Parkinson’s and Movement Disorders Society Rare Movement Disorders Study Group provides updates on rare movement disorders.

**Methods:**

In this narrative review, we discuss the differential diagnosis of the rare disorders that can cause chorea.

**Results:**

Although the most common causes of chorea are hereditary, it is critical to identify acquired or symptomatic choreas since these are potentially treatable conditions. Disorders of metabolism and mitochondrial cytopathies can also be associated with chorea.

**Discussion:**

The present review discusses clues to the diagnosis of chorea of various etiologies. Authors propose algorithms to help the clinician in the diagnosis of these rare disorders.

## Introduction

**Figure 1:**
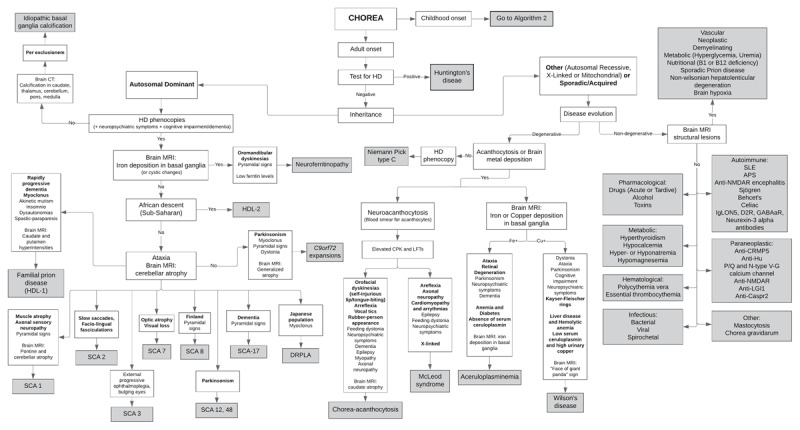
**Adult-onset choreas**. HD: Huntington disease; HDL: Huntington-disease like; SCA: spinocerebellar ataxia; DRPLA: dentatorubropallidoluysian atrophy; SLE: systemic lupus erythematosus; APS: antiphospholipid syndrome; NMDAR: n-methyl-d-aspartic acid receptor. Adapted from: Hermann A, Walker RH. Diagnosis and treatment of chorea syndromes. Current neurology and neuroscience reports 2015;15:514.

**Figure 2:**
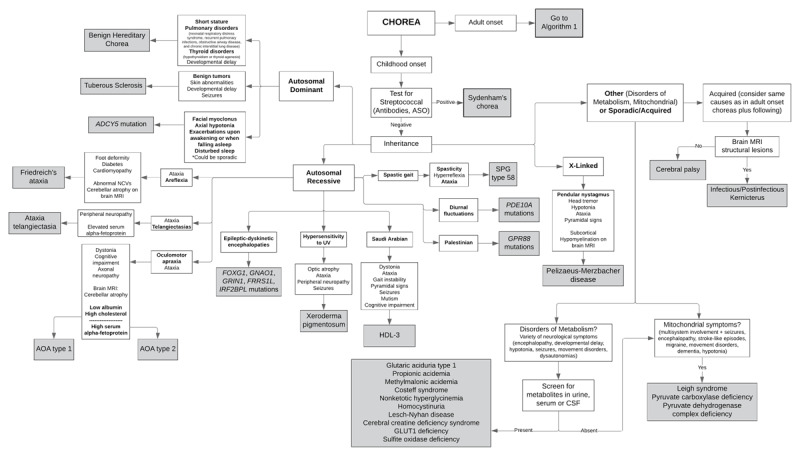
**Early/Childhood-onset choreas**. HDL: Huntington-disease like; AOA: ataxia with oculomotor apraxia; ASO: antistreptolysin O; SPG: spastic paraplegia. Adapted from: Hermann A, Walker RH. Diagnosis and treatment of chorea syndromes. Current neurology and neuroscience reports 2015;15:514.

Accurate diagnosis of rare movement disorders (RMD) is often challenging. Although different strategies for approaching RMD have been suggested, including identification of red flags, syndromic patterns, or genetic findings, none are highly reliable. One of the main objectives of the Rare Movement Disorders Study Group of the International Parkinson and Movement Disorders Society is to educate and help clinicians better diagnose RMD. Because of the large number of RMD, many of which have overlapping phenotypes, we proposed an initial approach according to the predominant movement disorder in the clinical evaluation. The present review provides an update on the differential diagnosis of rare disorders that can cause choreas. We have included disorders where chorea is predominant in the clinical picture. We also propose algorithms to guide clinicians in the diagnosis of these rare disorders (Figures 1 and 2).

## Recommended Approach

The first step in diagnosis is recognizing the movement disorder as chorea [1]. Chorea is characterized by irregular, purposeless, arrhythmic, non-stereotyped involuntary movements that flow from one body part to another [2]. These movements can affect any part of the body, although usually brief, can also be of long duration, and can have small or large amplitude. Movements with large amplitude localized proximally in the arms are called “ballismus” [3].

Once the movement disorder has been recognized as chorea, we then recommend classifying the age of onset as either childhood- (prior to the age of 16) or adult-onset [1]. Multiple causes of chorea exist in both age groups, many of which fit in the definition of rare diseases [4]. Since the most common cause of acute chorea in childhood is Sydenham’s chorea and of chronic progressive chorea in adults is Huntington’s disease (HD), we recommend these disorders should be tested for, as appropriate, and excluded before proceeding to other diagnostic pathways.

The next step is to determine, if possible, whether the chorea is hereditary (genetic) or acquired (non-genetic) in nature. The family history should be carefully and systematically evaluated; the absence of a family history does not exclude a hereditary cause. Disorders of metabolism and mitochondrial cytopathies are also cause of hereditary choreas. The diagnosis of chorea can be challenging and even with an extensive work-up, some patients remaining undiagnosed. Next generation sequencing technologies have helped increase the candidate gene list associated with choreas and other movement disorders, facilitating the subsequent diagnosis and understanding the disease process [5,6]. However, this field is in constant evolution with new genes being discovered each year as cause of chorea. It is, of course, critical to identify acquired causes of chorea since these are potentially treatable conditions. The main causes of acquired choreas include infectious/post-infectious, autoimmune, metabolic, vascular, and drug-induced causes. Other less common causes of acquired choreas include posttraumatic, paraneoplastic, or demyelinating conditions. The present review includes possible etiologies of rare disorders where chorea is a predominant symptom in the clinical picture.

The following sections are organized by possible etiologies followed by a brief discussion of diagnostic clues for the different diseases. We also provide algorithms to offer the clinician a guide to organize their diagnostic thinking about chorea. Readers need to be aware that this algorithmic method cannot account all possible scenarios and should be interpreted within the clinical context.

## Hereditary Choreas

Hereditary choreas are inherited in autosomal dominant, autosomal recessive, and X-linked hereditary modes. The hereditary choreas are described in Table 1. The paroxysmal kinesigenic dyskinesias are beyond the scope of this review.

**Table 1a:** Characteristics of adult-onset autosomal dominant hereditary choreas.

Disease; *Gene*	Region; Age	Mov Disord	Key Findings	Other Sx	NPsych	CI/Dem	I and L

Huntington’s disease; *Huntingtin (HTT)*	20–40 yrs.*	Chorea Parkinsonism, dystonia, myoclonus if young onset	–	Hung-up knee jerk reflex	Yes	Yes	I: Striatal volume loss
C9orf72 expansions; *C9orf72*	Caucasian; 40–50 yrs.	HD phenocopy	–	–	Yes	Yes	I: Generalized cerebral atrophy
Spinocerebellar ataxia 17; *TATA box-binding protein (TBP)*	Caucasian and Asian; 20–40 yrs.	HD phenocopy Dystonia, tremors, parkinsonism	Ataxia predominates	Pyramidal signs Seizures	Yes	Yes	I: caudate or cerebellar atrophy
Huntington disease-like 2; *Junctophilin-3 (JPH3)*	African ancestry; 40–50 yrs.	HD phenocopy	–	–	Yes	Yes	I: Similar to HD
Dentatorubropallidoluysian atrophy; *Atrophin-1 (ATN1)*	Japanese; 30 yrs.	HD phenocopy Myoclonus	–	Ataxia Seizures	Yes	Yes	I: White matter lesions and cerebellar/brainstem atrophy
Neuroferritinopathy; *Light chain of ferritin (FTL)*	Cumbrian region of northern England; 40 yrs.	HD phenocopy Dystonia, parkinsonism	Oromandibular chorea predominate	Spasticity Ataxia	Yes	Yes	I: Iron accumulation or cystic changes in basal ganglia or cortical regions with pallidal necrosis and edema in later stages L: Low serum ferritin levels
Familial prion disease (Huntington disease-like 1); *Prion protein (PRNP)*	20–40 yrs.	HD phenocopy Myoclonus	Rapidly progressive	Seizures Ataxia	Yes	Yes	–
Spinocerebellar ataxias types 1, 2, 3, 7, 12, 48; *ATXN 1, 2, 3, 7, 12, 48*	European, Cuban, Indian (SCA 2); 30 yrs.*	HD phenocopies Dystonia, myoclonus, parkinsonism	Ataxia	Pyramidal signs Oculomotor abnormalities Slow saccades (SCA2) Facio-lingualfasciculations (SCA2)	No	Yes	I: Pontine and cerebellar atrophy; T2 hyperintensities in dentate nuclei extending to middle cerebellar peduncle (SCA48)
Primary familial brain calcification; *SLC20A2*	30–40 yrs.	Akinetic syndrome, tremor, chorea, dystonia	–	–	Yes	Yes	I: Caudate, brainstem, thalami, cerebellum, white matter, cortical calcifications

Mov Dis: Movement Disorders; Sx: Symptoms; NPsych: Neuropsychiatric features; CI/Dem: Cognitive impairment or dementia; I: Imaging; L: Laboratory; HD: Huntington’s disease. * Inversely related to the size of the repeat expansion

**Table 1b:** Characteristics of adult-onset autosomal recessive hereditary choreas.

Disease; *Gene*	Region; Age	Mov Disord	Key Findings	Other Sx	NPsych	Ci/Dem	I and L

Chorea-acanthocytosis; *VPS13A*	20–40 yrs.	Generalized chorea, dystonia, tics, parkinsonism	Self-injurious orofacial dyskinesia Rubber-person-like gait	Sp & Sw and problems Feeding dystonia Hypersalivation Head drops Myopathy or neuropathy Seizures	Yes	Yes	I: Caudate atrophy L: Acanthocytes on blood smear, CPK and LFT elevation
Aceruloplasminemia; *CP*	40–50 yrs.	Chorea Parkinsonism	Retinal degeneration	Ataxia	Yes	Yes	I: Symmetrical iron deposition in the basal ganglia, thalamus, red nuclei, and dentate nuclei L: Anemia, DM
Wilson’s disease; *ATP7B*	6–50 yrs.	Dystonic/choreic syndrome Parkinsonian syndrome	Kayser-Fleischer rings	Ataxic syndrome	Yes	Yes	I: “Face of the giant panda” in midbrain L: Low serum ceruloplasmin levels, Elevated 24-hours copper excretion, Hemolytic anemia, Elevated LFTs

Mov Dis: Movement Disorders; Sx: Symptoms; NPsych: Neuropsychiatric features; CI/Dem: Cognitive impairment or dementia; I: Imaging; L: Laboratory; Sp: Speech; Sw: Swallowing.

**Table 1c:** Characteristics of childhood-onset autosomal dominant hereditary choreas.

Disease; *Gene*	Region; Age	Mov Disord	Key Findings	Other Sx	NPsych	CI/Dem	I and L

ADCY5 mutation; *ADCY5*	0–20 yrs.	Chorea, myoclonus, dystonia	Episodic exacerbations of dyskinesia upon awakening or when falling asleep	Delayed milestones and axial hypotonia	No	No	–
Benign Hereditary Chorea; *NKX2-1*	1–20 yrs.	Chorea with mild progression	Pulmonary and thyroid problems	Short stature Developmental delay	No	No	–
Tuberous sclerosis; *TSC1* and *TSC2*	10 yrs.	Chorea	Benign tumors Skin abnormalities	Behavioral symptoms Seizures Developmental problems	Yes	Yes	I: Subependymal nodules and cortical/subcortical tubers

Mov Dis: Movement Disorders; Sx: Symptoms; NPsych: Neuropsychiatric features; CI/Dem: Cognitive impairment or dementia; I: Imaging; L: Laboratory.

**Table 1d:** Characteristics of childhood-onset autosomal recessive hereditary choreas.

Disease; *Gene*	Region; Age	Mov Disord	Key Findings	Other Sx	NPsych	CI/Dem	I and L

Friedreich’s ataxia; *Frataxin*	10–15 yrs.	Chorea is rare	Ataxia Foot deformities Areflexia	Peripheral neuropathy Diabetes Cardiomyopathy Scoliosis	No	No	NCS: Abnormal sensory nerve action potentials I: Atrophy of cervical spinal cord with minimal cerebellar atrophy
Ataxia telangiectasia; *ATM*	1–4 yrs.	Choreoathetosis	Prominent ataxia Telangiectasias	Peripheral neuropathy Oculomotor apraxia Predisposition to immunological disorders and cancer	No	No	I: cerebellar atrophy L: Serum alpha-fetoprotein is elevated and IgA, IgE, IgG2 deficiency
Ataxia with oculomotor apraxia types 1 and 2; *APTX* and *SETX*	2–10 yrs. (AOA1) 3–30 yrs. (AOA2)	Chorea, dystonia	Oculomotor apraxia Ataxia	Axonal sensorimotor neuropathy	No	Yes	I: Cerebellar atrophy L: Hypoalbuminemia and Hypercholesterolemia (AOA1), Elevated serum alpha-fetoprotein (AOA2)
Xeroderma pigmentosum; *XP*	1–2 yrs.	Chorea in advanced stages	Cutaneous photosensitivity Freckle-like pigmentation on face Conjunctival telangiectasia Optic atrophy Dry skin Predisposition to skin cancer	Ataxia Seizures Areflexia Peripheral neuropathy Sensorineural hearing loss Abnormalities in dentition	No	Yes	–
Huntington disease-like 3; unknown	Saudi Arabian; 3–4 yrs.	Chorea, dystonia	–	Ataxia Gait instability Spasticity Seizures Mutism	No	Yes	I: Frontal and caudate atrophy
Spastic ataxia type 2 (SPG58); *KIF1C*	10–20 yrs.	Chorea Dystonia	Spastic gait Ataxia	Hyperreflexia and spasticity Developmental delay	No	Yes	I: Demyelination and cerebellar atrophy
PDE10A mutation; *PDE10A*	5–15 yrs.	Chorea	Diurnal fluctuations	–	No	No	I: Symmetrical T2-hyperintense striatal lesions
GPR88 mutation; *GPR88*	Palestinian; 8–9 yrs.	Chorea	–	Developmental delay	No	Yes	–
Hereditary Epileptic-Dyskinetic Encephalopathies; *FOXG1, GNAO1, GRIN1, FRRS1L, IRF2BPL*	Neonatal to 8 yrs.	Chorea, dystonia, ballism, stereotypies	Early-onset, drug-resistant seizures Facial and oro-lingual dyskinesias	Developmental delay Oculogyric crises, cortical blindness, spastic tetraparesis (*GRIN1*)	No	Yes	I: corpus callosum hypoplasia, delayed myelination, simplified gyration (*FOXG1*)

Mov Dis: Movement Disorders; Sx: Symptoms; NPsych: Neuropsychiatric features; CI/Dem: Cognitive impairment or dementia; I: Imaging; L: Laboratory.

**Table 1e:** Characteristics X-linked hereditary choreas.

Disease; *Gene*	Region; Age	Mov Disord	Key Findings	Other Sx	NPsych	CI/Dem	I and L

McLeod syndrome; *XK*	Before 40 yrs.	Chorea, dystonia, parkinsonism	Areflexia Sensorimotor axonopathy	Cardiomyopathy Arrhythmias MyopathySeizures	Yes	No	L: Elevated serum liver enzymes and creatine kinase I: caudate nucleus and putamen atrophy
Pelizaeus-Merzbacher disease; *PLP1*	Neonatal to 5 yrs.	Choreoathetosis Dystonia	Pendular nystagmus Spasticity Head tremor Generalized hypotonia	Ataxia Mental retardation	No	Yes	I: Hypomyelination of corona radiata, optical radiations, internal capsule

Mov Dis: Movement Disorders; Sx: Symptoms; NPsych: Neuropsychiatric features; CI/Dem: Cognitive impairment or dementia; I: Imaging; L: Laboratory

### 1. Autosomal Dominant

#### HD phenocopies

When the family history and clinical presentation points to HD but genetic testing is negative, as is reported in 1–3% of cases, clinicians should consider other autosomal dominant disorders that are phenotypically similar to HD, so-called HD phenocopies. These HD phenocopies include neurodegenerative diseases caused by repeat expansions in the *C9orf72* gene, spinocerebellar ataxia (SCA) 17, Huntington disease-like 2 (HDL2), dentatorubropallidoluysian atrophy (DRPLA), neuroferritinopathy, familial prion disease (e.g. Huntington disease-like 1 [HDL1]) [7]. Recently, mutations in new genes associated with HD phenocopy syndromes have been identified, including *CACNA1A, VSP13A*, and *VCP* [8,9].

##### C9orf72 expansions

*C9orf72* expansions are a rare cause of chorea, but appear to be the most common cause of HD phenocopies in Caucasian populations [10]. The expanded hexanucleotide repeat in *C9orf72* gene is responsible for diseases such as amyotrophic lateral sclerosis and frontotemporal lobar dementia [11]. Patients typically present around 40–50 years of age, and although a variety of movement disorders and neuropsychiatric symptoms may develop, the clinical features may be quite similar to those of HD [12]. Upper motor neuron abnormalities and frontal lobe signs may suggest the diagnosis.

##### Spinocerebellar ataxia 17 (SCA17)

SCA17 has been reported in Caucasian and Asian populations [13]. A family originally described with what was termed “Huntington’s disease-like 4” was subsequently diagnosed with this disorder. Patients with SCA17 are characterized by a clinical picture dominated by ataxia, in addition to other movement disorders such as chorea, dystonia, tremors, or parkinsonism. Patients may also develop pyramidal signs, cognitive impairment, seizures, or psychiatric symptoms. Age of onset is variable, but usually presents in early to mid-life (between 20s–40s) [14]. SCA17 is caused by a trinucleotide CAG repeat expansion of chromosome 6q27 of the *TATA box-binding protein* (*TBP*) gene. Brain MRI typically shows caudate nucleus or cerebellar atrophy while putaminal rim hyperintensities have rarely been reported [15].

##### Huntington disease-like 2 (HDL2)

HDL2 has only been reported in people of African ancestry, and has been documented primarily in South Africa, in addition to North, Central and South America, the Caribbean, and Europe [16,17]. HDL2 is the most commonly reported HD phenocopy in this population. Fewer than 100 cases have been reported in the literature worldwide. HDL2 strongly resembles HD clinically, radiologically, and neuropathologically [18]. HDL2 is caused by a CAG repeat expansion of the *junctophilin-3* (*JPH3*) gene on chromosome 16q24.2 [17,19]. As with HD, the age of onset is inversely related to the size of CAG repeat expansion.

##### Dentatorubropallidoluysian atrophy (DRPLA)

DRPLA is characterized by a striking variety of symptoms including seizures, ataxia, chorea, myoclonus, dementia, and psychiatric symptoms that vary with age of presentation. Some patients can present with a clinical phenotype very similar to that of HD, with chorea as the predominant manifestation. DRPLA is highly prevalent among the Japanese population, but has occasionally been reported elsewhere [20]. The age of presentation depends upon the size of the CAG repeat expansion in *atrophin-1* (*ATN1*) on chromosome 12p13–31 [21]. Brain MRI typically shows white matter lesions and cerebellar and brainstem atrophy.

##### Neuroferritinopathy

Neuroferritinopathy is a “neurodegeneration with iron accumulation (NBIA)” disorder, characterized by a variety of movement disorders including chorea, dystonia, and parkinsonism. A clinical hallmark is the presence of prominent oromandibular chorea. Patients can develop other neurological symptoms such as dysarthria, spasticity, cerebellar signs, frontal lobe symptoms, or dementia. Symptoms of neuroferritinopathy usually develop around the age of 40; the diagnosis can be made by the presence of very low serum ferritin levels [22]. Brain MRI shows iron accumulation or cystic changes in cortical regions or in the basal ganglia with pallidal necrosis and edema in later stages of the disease [23]. Muscle biopsy may show abnormalities in the mitochondrial respiratory chain. Neuroferritinopathy is exceedingly rare, and has been reported predominantly in the Cumbrian region of England. It is caused by a mutation on chromosome 19q13 of the *light chain of ferritin* (*FTL*) gene.

##### Prion diseases

The majority of the cases of prion disease are either sporadic or acquired in nature [24].

Around 15% of all prion disease are caused by mutations in the *prion protein* gene, which are inherited in an autosomal dominant fashion. Huntington disease-like 1 (HDL1) is a rare familial prion disease with which can rarely present with similar clinical manifestations to HD, in addition to seizures and ataxia [25]. Prominent psychiatric symptoms and myoclonus can suggest this diagnosis. More typically, it causes cognitive problems, neuropsychiatric symptoms, or ataxia; chorea is rare. Age of presentation is similar to that of HD in early adulthood, between the 20s and 40s. Symptoms lead to death within months or years. Neuropathology typically shows basal ganglia and frontotemporal and cerebellar atrophy with multicentric plaques that stain with anti-prion antibodies. HDL1 is caused by a mutation on chromosome 20p12 of the *prion protein* (*PRNP*) gene [26]. Of the acquired/sporadic prion disorders, a new variant Creutzfeldt-Jakob disease (associated with Bovine Spongiform Encephalopathy) has been reported to cause chorea [27].

##### Spinocerebellar ataxias

Other spinocerebellar ataxias, such as SCA1, SCA2, SCA3, SCA7, SCA8, SCA12, and SCA48 should be considered in the differential diagnosis of autosomal dominant hereditary choreas with an ataxic phenotype [28]. Trinucleotide repeat expansion disorders, as in these SCAs, display the anticipation phenomenon. Many of the SCAs are caused by mutation of their respective *ataxin* (ATXN) genes. SCA1 has a mean age of onset in the 30s. Fifteen percent of the patients will develop chorea. In addition to ataxia, patients may develop pyramidal symptoms, dystonia, or oculomotor abnormalities. Brain MRI shows pontine and cerebellar atrophy [29]. SCA2 is a common reported cause of HD phenocopies in European populations, Cuban and Indian ethnicities. The average age of onset is similarly in the 30s. Chorea may be present, however, additional typical findings in SCA2 are impaired slow saccades, myoclonus, facio-lingual fasciculations, cognitive impairment, and parkinsonism. Brain MRI also shows pontine and cerebellar atrophy [30]. SCA3, also known as Machado-Joseph disease, is the most common autosomal dominant ataxia worldwide. It has a wide range of onset ages and a variety of clinical manifestations. Brain MRI also shows pontine and cerebellar atrophy [31]. SCA7 presents typically with ataxia, visual loss, ophthalmoplegia, and rarely chorea. Brain MRI shows cerebellar and brainstem atrophy [28,32]. SCA8 is highly prevalent in Finland and its clinical presentation is highly variable, however, reports describe several symptoms that seem to be share between cases, including ataxia, pyramidal symptoms, sensory symptoms, cognitive impairment, myoclonus, and migraine headaches. MRI shows cerebellar atrophy [33]. Chorea can be an atypical clinical characteristic of SCA8 [34]. SCA12 is characterized by slowly progressive ataxia, neuropsychiatric symptoms, and rarely with cognitive decline. Additional features are parkinsonism and hyperreflexia. Recently, a case was reported to present as HD-like, expanding the phenotypic spectrum of SCA12. Brain MRI shows cerebral cortex atrophy [35]. SCA48 has recently been described as an adult-onset ataxia associated with a cognitive-psychiatric disorder and other variable symptoms including chorea, parkinsonism, dystonia, epilepsy, and urinary problems. MRI shows cerebellar atrophy and T2 hyperintensities in the dentate nuclei extending to middle cerebellar peduncles [36].

#### Non-HD phenocopies

##### Primary Familial Brain Calcification

Primary familial brain calcification (PFBC) is a neurodegenerative disorder characterized by calcium deposits in a variety of brain areas observed on neuroimaging. Brain CTs show calcification in caudate nuclei, cerebellum, white matter, thalami, cortex, vermis, midbrain, pons, and medulla. PFBC is predominantly caused by mutations in *SLC20A2* gene, although a number of other genes have recently been implicated, such as *PDGFB, PDGFRB*, and *XPR1* genes [37]. The pattern of inheritance is an autosomal dominant pattern in most cases. The clinical symptoms are variable, and can include cognitive impairment, psychiatric symptoms, and movement disorders. The age of onset is usually in the 30s and 40s. The most common movement disorder reported is an akinetic syndrome with or without tremor, although chorea and dystonia can also be seen [38].

##### ADCY5-Related Dyskinesia

Mutations in *ADCY5* were first reported in families described as having “Familial Dyskinesia with Facial Myokimia” [39]. The clinical phenotype has expanded since then; core features include infantile or early childhood-onset of facial myoclonus, axial hypotonia, and exacerbations of dyskinetic movements in an episodic manner in relation to drowsiness and sleep [40]. The dyskinetic movements have been variably described as chorea, athetosis, dystonia, or myoclonus, typically more prominent in the upper limbs [40]. It is important to note that while *ADCY5* mutations are typically transmitted in autosomal dominant fashion, it also can occur *de novo* [41]. Delayed milestones and signs of spasticity may also be part of the clinical spectrum. *ADCY5* mutations have also been found in patients carrying the diagnosis of “benign hereditary chorea” [42].

##### Benign Hereditary Chorea

Mutations in *TITF1/NKX2-1* are the most common cause of benign hereditary chorea (BHC) [43]. BHC typically presents in childhood, preceded by hypotonia, and is characterized by chorea with mild clinical progression [44]. It is argued that this is not necessarily a “benign” condition, as it is associated mainly with pulmonary and thyroid disorders in the most severe expression of the TITF1/NKX2-1-related disorders. This triad is also called “brain-lung-thyroid disease” [45]. Commonly reported pulmonary disorders include neonatal respiratory distress syndrome, recurrent pulmonary infections, obstructive airway disease, and chronic interstitial lung disease. Thyroid disorders reported may include congenital hypothyroidism or thyroid agenesis [46]. Mutations in *SLC16A2* gene have also been described as a cause of BHC or “brain-lung-thyroid disease” [47]. Carriers of mutations in *TITF1/NKX2-1* can have increased risk of malignancy, specifically lung cancer, and should undergo appropriate screening [43].

##### Tuberous sclerosis

Tuberous sclerosis is a genetic disorder characterized by numerous benign tumors in many parts of the body, skin abnormalities, developmental problems, behavioral symptoms, and seizures. Although rare, chorea may be a manifestation of this complex disorder, likely related to nodules located in the basal ganglia [48,49].

### 2. Autosomal Recessive

#### Chorea-acanthocytosis

Chorea-acanthocytosis is one of the core neuroacanthocytosis syndromes, and is a progressive neurologic disorder characterized by prominent self-injurious orofacial dyskinesia and generalized chorea. Patients typically develop speech and swallowing problems, hypersalivation, or vocal tics such as grunts, snorts, echolalia, and other utterances. Feeding dystonia, in which the tongue pushes food out of the mouth during eating, is characteristic. Head drops can often be seen. Other neurological characteristics are neuropsychiatric symptoms, axonal neuropathy, seizures, and a gait which is described as “rubber-person”. The age of onset is usually in the third or fourth decades of life. Brain MRI shows caudate atrophy. Acanthocytes are seen on peripheral blood smear, although this is a variable finding. More useful for diagnosis is the observation that creatine kinase is elevated in the thousands (normal 0–200 U/I), reflecting muscle damage. Chorea-acanthocytosis is caused by mutations of the *VPS13A* gene on chromosome 9q21 [50].

#### Friedreich’s ataxia

Friedreich’s ataxia is characterized by progressive ataxia and peripheral neuropathy in patients under 25 years of age [51]. The disease is mainly caused by a homozygous GAA triplet repeat expansion in the *frataxin* (*FXN*) gene; a shorter repeat expansion length correlates with older age at onset and milder disease [52]. Around 2% of the patients are compound heterozygotes who have repeat expansions in one allele with a point mutation in the other allele [53]. Patients with heterozygous repeat expansion may have atypical clinical features, such as chorea. However, chorea has also been reported in patients homozygous for the expansion [54].

#### Aceruloplasminemia

Aceruloplasminemia is a disorder of iron metabolism, considered as one of the NBIA disorders, that shares clinical characteristics with HD. Patients tend to develop anemia and diabetes in their 20s and may present with ataxia, chorea, parkinsonism, cognitive decline, and psychiatric symptoms. Retinal degeneration is a characteristic feature. Brain MRI displays symmetrical deposition of iron in the basal ganglia, thalamus, red nuclei, and dentate nuclei. Aceruloplasminemia is caused by mutations in the *CP* gene [55].

#### Huntington disease-like 3 (HDL3)

Only one Saudi Arabian family consisting of 5 affected individuals was reported to have a disease termed “HDL3”. The age of onset was in childhood (3–4 years of age) with a variety of clinical manifestations such as chorea, dystonia, ataxia, gait instability, spasticity, seizures, mutism, and intellectual impairment. Brain MRI shows frontal and caudate atrophy. The causative gene is still unknown; the disease locus mapped to chromosome 4p15.3 [56], although this has been questioned.

#### Wilson’s disease

Wilson’s disease typically presents in the second or third decade of life, is a disorder of copper transport leading to copper accumulation, and is one of the few potentially treatable hereditary movement disorders [57]. The neurological manifestations vary widely. Three distinct neurological presentations are suggested; a dystonic/choreic syndrome, an ataxic syndrome, and a parkinsonian syndrome. Chorea is reported in 9% of Wilson patients and should therefore be considered in the differential diagnosis of HDL disorders. Patients also develop cognitive impairment and neuropsychiatric symptoms. Evidence of liver disease and hemolytic anemia should prompt the clinician to consider the diagnosis, which is supported by low ceruloplasmin levels, elevated 24-hour copper excretion, and Kayser-Fleischer rings on slit-lamp ophthalmological examination. Abnormal signals in the putamen, caudate nucleus, globus pallidus, thalamus are frequently seen on brain MRI in addition to brainstem changes [58]. Although not uniformly present across patients, brain MRI shows a characteristic “face of the giant panda” in the midbrain. Wilson’s disease is caused by mutations in *ATP7B* gene [59]. The goal of therapy is to establish a net negative copper balance. This can be achieved by increasing copper excretion with chelating agents, and by reducing copper absorption with zinc and reducing dietary intake [57].

#### Ataxia-telangiectasia

Ataxia-telangiectasia is characterized by progressive neurological dysfunction associated with multisystem abnormalities and predisposition to immunological disorders and cancer. Chorea is commonly seen in ataxia-telangiectasia syndrome, with onset in childhood, prominent ataxia, peripheral neuropathy, and telangiectasias (conjunctiva and external ear). The serum concentration of alpha-fetoprotein is elevated. In addition, patients develop immunoglobulin deficiency resulting in repetitive upper respiratory tract infections, and have increased sensitivity to radiation with high risk of developing cancers. Ataxia-telangiectasia is caused by mutations in *ATM* gene [60].

#### Ataxia with oculomotor apraxia 1 and 2

Ataxia with oculomotor apraxia 1 (AOA1) typically presents with ataxia, axonal sensorimotor neuropathy, oculomotor apraxia, and movement disorders including chorea and/or dystonia. Ataxia with oculomotor apraxia 2 (AOA2) presents similarly to AOA1 [61]. Cognitive impairment is more frequent in AOA1 than in AOA2. AOA1 patients have hypoalbuminemia and hypercholesterolemia, in contrast with AOA2 patients who have elevated serum alpha-fetoprotein levels. The age of onset is usually earlier in AOA1 compared to AOA2, which presents during childhood and adolescence. Brain MRI in both AOAs usually shows cerebellar atrophy. AOA1 is caused by mutations in *APTX* gene and AOA2 is caused by mutations in *SETX* gene.

#### Spastic ataxia type 2/Spastic paraplegia type 58

The hereditary spastic paraplegias (HSPs) are a group of heterogeneous disorders characterized by spastic gait, hyperreflexia, and spasticity. Complex HSPs are associated with additional neurologic manifestations including ataxia and other movement disorders. Chorea has been reported in spastic paraplegia type 58, also known as spastic ataxia type 2, caused by a mutation in *KIF1C* gene. The age of onset ranges from infancy to adulthood [62].

#### Xeroderma pigmentosum (XP)

XP is caused by mutations in the nucleotide excision repair pathway genes causing cutaneous, ocular and neurological manifestations. The cutaneous signs usually appear in infancy or early childhood. The neurological manifestations typically occur after the cutaneous symptoms. Movement disorders, specifically chorea, may appear in advanced stages of the disease. Patients with dermatological and oncological manifestations should be screened for XP [63].

#### Hereditary Epileptic-Dyskinetic Encephalopathies

The epileptic-dyskinetic encephalopathies are clinically heterogenous and are characterized by early onset drug-resistant seizures associated with hyperkinetic movement disorders. When chorea is the dominant movement disorder, mutations in *FOXG1, GNAO1, GRIN1, FRRS1L* and *IRF2BPL* genes should be considered [64,65].

#### PDE10A mutation

Mutations in *PDE10A* have been reported to be inherited in both dominant and recessive manners; patients present with early-onset (5–15 years of age) chorea. Cognition and development is typically normal, however, those carrying biallelic mutations show a more severe phenotype. Brain MRI may show symmetrical T2-hyperintense bilateral striatal lesions [64].

#### GPR88 mutation

Mutations in *GPR88* has been reported in three children of one consanguineous Palestinian family. Affected individuals with *GPR88* mutation typically present with early onset slowly progressive chorea, mental retardation, and developmental delay [64].

### 3. X-linked

#### McLeod syndrome

McLeod syndrome is an X-linked recessive hereditary disorder with very similar clinical characteristics to chorea-acanthocytosis. Patients lack the red blood cell XK antigen and the expression of Kell antigens on the erythrocyte membrane surface is reduced. Chorea is a common symptom in addition to areflexia due to a sensorimotor axonopathy. Distinguishing features are male gender, the age of onset, which tends to be in the 40s–60s, and the presence of cardiomyopathy and arrhythmias. Liver enzymes and creatine kinase are elevated. Patients also develop seizures, significant myopathy, and peripheral neuropathy. While less common than in chorea-acanthocytosis, there can sometimes be tongue protrusion, feeding dystonia, and lip-biting or tongue-bitting [25,66].

#### Pelizaeus-Merzbacher disease

Pelizaeus-Merzbacher disease (PMD) is a clinically and genetically heterogeneous leukodystrophy characterized by central hypomyelination with neurologic dysfunction and progressive deterioration [67]. PMD usually presents within the first years of life with pendular nystagmus, head tremor, generalized hypotonia, mental retardation, choreoathetosis, cerebellar ataxia, and pyramidal signs. Brain MRI shows hypomyelination of the corona radiata, optical radiations, and internal capsule. PMD is caused by mutations in the *PLP1* gene which encodes the proteolipid protein of myelinating oligodendroglia.

### 4. Inborn Errors of Metabolism

Inborn errors of metabolism are common causes of hyperkinetic and hypokinetic movement disorders in children. A single error of metabolism can cause multiple movement disorders. Diagnosis of these disorders requires screening for specific metabolites in urine, serum or cerebrospinal fluid, the results of which can direct further biochemical or genetic analyses. Movement disorders in these children can cause life-long disability. Chorea is not an uncommon symtptom of inborn errors of metabolism (Table 2), and is a predominant characteristic of glutaric aciduria type 1, glucose transporter type 1 (GLUT1) deficiency, and Lesch-Nyhan disease. A recent report described that Niemann Pick type C in the adult-onset can mimic HD phenocopies and should be considered in the diagnostic approach of patients with a choreic phenotype [68].

**Table 2:** Characteristics of inborn errors of metabolism and mitochondrial cytopathies where chorea is the predominant movement disorder.

Type of Metabolic Disorder	Conditions; *Gene*	Region; Age	Inheritance Pattern	Mov Disord	Key Findings	Seizures	Ataxia	Pyramidal signs	DD	Other Sx	I and L	Tx

Organic acidemias	Glutaric aciduria type 1; *GCDH*	Neonatal	AR	Choreo-athetosis Dystonia Orofacial dyskinesia	Macrocephaly Acute encephalopatic crisis Opisthotonus	Yes	No	No	Yes	No	L: urinary glutaric acid, 3-hydroxyglutaric acid, glutaconic acid.	Low lysine diet and oral carnitine supplementation
	Propionic acidemia; *PCCA* and *PCCB*	Amish, Inuit of Greenland and Saudi Arabian populations; Neonatal or infancy	AR	Choreoathetosis	Poor feeding Lethargy Encephalopathy Vomiting Hypotonia	Yes	No	Yes	Yes	Hepatomegaly Failure to thrive Optic atrophy Hearing loss Premature ovarian failure Chronic renal failure Cardiomyopathy Attention-deficit disorder Autism	L: Plasma amino acids, acylcarnitines, and urinary organic acids, and orotic acid. I: Lesions in the bilateral lenticular and caudate nuclei	Protein-restricted diet; Treat metabolic acidosis, hypoglycemia, hyperammonemia
	Methylmalonic acidemia; *MUT*	First year	AR	Choreoathetosis Dystonia	Encephalopathy Stroke Hypotonia Lethargy Monilial infections	Yes	No	No	Yes	Dysphagia Dysarthria Failure to thrive Hepatosplenomegaly	L: Elevated blood and urine levels of ammonia, glycine, methylmalonic acid, propionic acid. I: Bilateral globus pallidus lesions	Protein-restricted diet, cyanocobalamin, and levo-carnitine supplementation
	OPA3-related 3-methylglutaconic aciduria (Costeff syndrome); *OPA3*	Iraqi-Jewish descent; Before age ten years	AR	Choreoathetosis	Optic atrophy Spastic paraparesis	No	Yes	No	No	No	L: Urinary excretion of 3-methylglutaconic acid	Supportive
Amino acid metabolism	Nonketotic hyperglycinemia; *GLDC* or *AMT*	Neonatal, infancy, adulthood	AR	Chorea	Lethargy, coma Hypotonia, hiccups Myoclonic jerks	Yes	No	Yes	Yes	Breathing/swallowing disorders	I: Elevated glycine levels in CSF and plasma.	Sodium benzoate Dextromethorphan Seizure management
	Homocystinuria; *CBS MTHFR, MTR, MTRR, MMADHC*	1st or 2nd decade	AR	Chorea Dystonia	Ectopia lentis, severe myopia Thromboembolism Skeletal and skin abnormalities	Yes	No	No	Yes	No	L: Increased serum levels of homocysteine and methionine	Vitamin B6 Methionine-restricted diet Folate Vitamin B12 Betaine
Purine metabolism	Lesch-Nyhan disease; *HPRT1*	3 to 6 mo.	X-linked	Choreoathetosis Dystonia	Self-injurious behavior Gouty arthritis Crystals or calculi in kidneys, ureters, bladder	No	No	Yes	Yes	Hypotonia Behavioral disturbances	L: Urinary urine-to-creatine ratio greater than 2.0 Hyperuricemia Hyperuricuria	Allopurinol Symptomatic
Creatine metabolism	Cerebral creatine deficiency syndrome 2 (GAMT deficiency); *GAMT*	Early infancy to 3 yrs.	AR	Choreoathetosis Dystonia	Hyperactivity, autism, self-injurious behavior	Yes	Yes	No	No	No	I: Hyperintensities in basal ganglia	Creatine monohydrate supplementation Ornithine supplementation Protein- or arginine-restricted diet
Glucose transport	GLUT1 deficiency; *SLC2A1*	Infancy	AD; AR	Chorea Dystonia	Paroxysmal episodes of mov disord or epilepsy Atypical childhood absence epilepsy Myoclonic astatic epilepsy	Yes	Yes	No	Yes	Microcephaly	L: Low CSF:serum glucose ratio	Ketogenic diet Symptomatic
Lipid storage	Niemann Pick Type C; *NPC1 or NPC2*	Infancy, children, adults	AR	HD phenocopy in adult onset Dystonia	Vertical supranuclear gaze palsy Dementia	Yes	Yes	No	Yes	Hypotonia Liver disease Respiratory failure	I: cerebellar atrophy or periventricular hyperintensities	Symptomatic
Other	Sulfite oxidase deficiency; *SUOX*	Infancy	AR	Choreoathetosis Dystonia	Ectopia lentis Eczema Failure to thrive	Yes	Yes	No	Yes	Hypotonia	L: Increase sulfite levels in urine I: Calcification of basal ganglia and cerebellar hypoplasia	Low sulfur amino acid diet Low protein diet
Mitochondrial cytopathies	Leigh syndrome and Leigh-like syndromes; *MT-ND, MTATP6*	First mo. to yrs. of life	X-linked, AR	Choreoathetosis Dystonia Parkinsonism	Ophthalmologic abnormalities Cardiac, hepatic, gastrointestinal and renal symptoms.	No	Yes	No	Yes	Hypotonia	L: Increased blood lactate levels. I: T2-weighted hyperintensities in basal ganglia and brainstem.	Biotin, thiamine, Coenzyme Q10 supplements
	Pyruvate carboxylase deficiency; *PC*	First yr.	AR	Choreoathetosis Tremors	Microcephaly Disconjugate eye movements, poor pupillary response, blindness, poor visual tracking Respiratory abnormalities	Yes	Yes	Yes	Yes	Hypotonia	L: Elevated blood levels of ammonia, pyruvate, lactate, acetoacetate and beta-hydroxybutyrate. I: periventricular WM cysts, subcortical FP hypernintensities.	Cofactor supplementation with thiamine and lipoic acid and administration of dichloroacetate
	Pyruvate dehydrogenase complex deficiency; *PDHA1*	Infancy	X-linked	Choreoathetosis Dystonia	Poor feeding Lethargy Tachypnea Abnormal eye movements Dysmorphic features Respiratory abnormalities	Yes	Yes	Yes	Yes	Microcephaly Hypotonia	L: Elevated blood levels of lactate and pyruvate I: absence of corpus callosum and medullary pyramids, ectopic inferior olives, symmetric cystic lesions and gliosis, generalized hypomyelination.	Cofactor supplementation with thiamine, carnitine, and lipoic acid.

Mov Dis: Movement Disorders; Sx: Symptoms; DD: Developmental delay; I: Imaging; L: Laboratory; AR: Autosomal recessive; AD: Autosomal dominant; CSF: cerebrospinal fluid; WM: white matter; FP: frontoparietal. * Only the disorders of metabolism where chorea is the predominant movement disorder are included. * Wilson’s disease is described under autosomal recessive hereditary choreas.

In general these disorders present in infancy or early childhood with a variety of neurological symptoms including encephalopathy, developmental delay, central or peripheral hypotonia, autonomic dysfunction, seizures and/or movement disorders [69]. Identification and management of potentially treatable inherited metabolic disorders is discussed elsewhere [70,71,72].

### 5. Mitochondrial Cytopathies

Mitochondrial disorders are a clinically and genetically heterogeneous group of diseases due to dysfunction of the mitochondrial respiratory chain, usually in the oxidative phosphorylation system and pyruvate dehydrogenase complex (Table 2) [73]. These disorders may present at any age with a highly variable clinical course and with a wide spectrum of clinical manifestations. Neurologic mitochondrial symptoms often include seizures, encephalopathy, stroke-like episodes, migraine, dementia, spasticity, and peripheral neuropathy. A multisystem clinical presentation involving several organs, including peripheral and central nervous systems should prompt the clinician to consider a possible mitochondrial disorder. A clue to the diagnosis is the presence of other family members with myopathic disorders or ophthalmoplegia [7]. Movement disorders are common in mitochondrial diseases. Patients with mutations in *POLG, MTTG, MTND4, HSD10, MICU1, COX20* have been reported to have choreic movements [74]. However, chorea is a predominant symptom in Leigh syndrome, pyruvate carboxylase deficiency, and pyruvate dehydrogenase complex deficiency [75]. The disorders of metabolism and mitochondrial cytopathies where chorea predominates are describe in Table 2.

## Acquired Choreas

In the following sections, we will discuss the rare acquired choreas (Table 3). We will not discuss some of the acquired choreas that are relatively common such as those induced by medications (levodopa-induced or tardive chorea), vascular choreas, Sydenham’s chorea, chorea secondary to non-ketotic hyperglycemia, neoplasms, causes of brain hypoxia, and cerebral palsy. Since a variety of genetic and metabolic conditions may mimic cerebral palsy, we recommend that the diagnosis of cerebral palsy be reconsidered when there is absence of risk factors in the birth/neonatal history or neuroimaging findings consistent with brain injury or congenital abnormalities [76].

**Table 3:** Causes of acquired choreas.

Etiology	Disease

Pharmacological	Acute drug-induced, Tardive chorea, Alcohol, Other toxins
Vascular	Stroke, Subdural or Extradural Hematomas, Small vessel disease
Hematological	Polycythemia vera, Essential thrombocythemia, Transitional myeloproliferative disease
Autoimmune	SLE, APS, NMDAR encephalitis, Behcet’s disease, Sjögren syndrome, Celiac disease, IgLON5, D2R, GABAaR, and Neurexin-3 alpha
Endocrine/Metabolic	Hyperthyroidism, Hypocalcemia, Hyper/Hyponatremia, Hyperglycemia, Hypomagnesemia, Uremia, Non-wilsonian hepatolenticular degeneration, Kernicterus
Nutritional	Vitamin B12 and B1 deficiency
Demyelinating disorders	Multiple Sclerosis, ADEM, Central pontine and extrapontine myelinolysis
Neoplastic	Primary or Secondary
Paraneoplastic	Anti-CRMP5, Anti-Hu, Anti-Ma, Anti-P/Q and N-type V-G calcium channel, Anti-NMDAR, Anti-LGI1, Anti-Caspr2
Infectious/Parainfectious	Sydenham’s chorea, Bacterial, Viral, Spirochetal
Brain hypoxia	Cardiac arrest, Respiratory insufficiency, Anesthetic complication, Hypothermia, CO Poisoning, Post pump chorea
Other	Mastocytosis, Chorea gravidarum, Cerebral palsy

SLE: systemic lupus erythematosus; APS: anti-phospholipid syndrome; NMDAR: anti-n-methyl-d-aspartate receptor; ADEM: acute disseminated encephalomyelitis.

### 1. Pharmacological

In addition to levodopa and dopamine agonists which are well-known to induce choreic movements in Parkinson’s disease patients, a long list of pharmacological agents with a wide spectrum of mechanisms of action has been reported to cause acute-onset chorea, including CNS stimulants (amphetamine and related drugs, methylphenidate and pemoline mesylate, cocaine), anticholinergics, antihistamines, antidepressants (tricyclics or SSRIs), opiates (methadone), anticonvulsants (phenytoin, carbamazepine, gabapentin, valproate, phenobarbitone, methobarbitone, primidone, methosuxamide, phenosuxamide, ethosuximide, beclamide, pheneturide, diazepam, sulthiame), lithium, oral contraceptives, baclofen, cimetidine, terbutaline, theophylline, anabolic steroids (oxymethalone), digoxin, amoxapine, fentanyl, cibenzoline, verapamil, and antibiotics (isoniazid, levofloxacin, cyclosporine).

#### Alcohol and other toxins

Chorea in alcohol abusers is more frequent in females and may be precipitated or exacerbated by withdrawal of alcohol. Movements are typically localized to the upper part of the body [77]. It is suggested that vitamin B1 deficiency contributes to the underlying cause [78]. Symptoms usually resolve within several weeks. Other toxins that may produce chorea include mercury, thallium, lead, and organophosphates [79].

### 2. Hematological

#### Polycythemia vera

Polycythemia vera (PCV) is a neoplastic bone marrow stem cell disorder characterized by an elevated red blood cell mass due to uncontrolled production of erythrocytes. Although a rare manifestation, gradual onset of generalized or asymmetric chorea can be a presenting symptom of PCV. Hypotonia and pendular or “hung-up” knee jerks have been reported. The underlying mechanism for the generation of chorea is uncertain, however hyperviscosity may lead to decreased blood flow in the basal ganglia. Patients are usually female and above 50 years of age, thus polycythemic chorea should be considered in the differential diagnosis of late-onset chorea, especially in women. In most cases the chorea is transient and self-limited, and responds to treatment of the polycythemia. Dopamine-blockers or -depleting agents are sometimes required [80].

#### Essential thrombocythemia

Essential thrombocythemia (ET) is a clonal myeloproliferative disease characterized predominantly by a markedly elevated platelet count without known cause, with predisposition to vascular occlusive events and hemorrhages. Generalized chorea has been reported recently as an initial presentation of ET [81].

### 3. Autoimmune

Factors that will guide the clinician to consider an autoimmune etiology of chorea are a subacute onset of the symptomatology, a fluctuating course with spontaneous remissions, frequent coexistence of neurological disorders atypical for HD, and absence of oculomotor abnormalities [82]. Sydenham’s chorea will not be discussed in the present review.

#### Systemic lupus erythematosus (SLE) and anti-phospholipid syndrome (APS)

The neurological system is involved in 20 to 65% of SLE cases. Patients may develop seizures, cranial nerve lesions, sensory abnormalities, neuropsychiatric symptoms, and neuropathies [83]. Chorea has been reported in about 4% of SLE patients, and in approximately 1/4 of these cases, chorea is the initial manifestation. Most cases are reported in young adult patients. The underlying mechanism associated with basal ganglia dysfunction remains to be determined, but potential pathophysiologies include vascular dysfunction as well as circulating antiphospholipid antibodies [84]. Some SLE patients are predisposed to develop APS. In most cases of SLE or APS, chorea is transitory. Cerebrospinal fluid analysis and brain MRI are usually normal. The treatment of chorea associated with SLE and APS relies on the combination of corticosteroids with or without other immunosuppressive agents, dopamine blockers or dopamine depletors. The use of aspirin or anticoagulants is recommended for APS patients [85].

#### Autoimmune Encephalitis with Antibodies against Plasma Membrane Proteins

##### Anti-N-Methyl-D-Aspartate receptor (NMDAR) encephalitis

Anti-NMDAR antibodies have recently emerged as an important cause of autoimmune encephalitis, which can result in a fatal outcome if not recognized and treated early [86,87]. This disorder affects young individuals (mean age at onset 21 years), predominantly female (4:1). Neuropsychiatric symptoms typically develop suddenly with an early presentation of movement disorders including chorea, stereotypies, and dystonia. Peri-oral movements are common. Cognitive disorders, autonomic symptoms, waxy flexibility, sleep problems, and seizures are also part of this complex disorder [88]. A significant number of cases are associated with underlying ovarian teratomas, with less frequent association of teratomas in men and children. Diagnostic criteria have been established [89]. Brain MRI is usually unremarkable and detection of CSF antibodies against the GluN1 subunit of the NMDAR confirms the diagnosis. Treatment includes immunosuppressive measures, corticosteroids, intravenous immunoglobulin (IVIg), plasma exchange, and in unresponsive cases, rituximab, cyclophosphamide, mycophenolate or other immunosuppressors are used [90]. The hyperkinetic disorders associated with anti-NMDAR encephalitis usually respond to dopamine depleting drugs, such as tetrabenazine, deutetrabenazine and valbenazine [84]. Anti-NMDAR encephalitis association with ovarian teratoma is common, especially in women of reproductive age. If present, surgical excision of teratoma is necessary to improve patient prognosis [91].

#### Behcet’s disease

Behcet’s syndrome is a multisystemic inflammatory disease characterized by ocular lesions (uveitis), genital and oral aphthosis, and skin lesions [92]. Neurological involvement is a rare manifestation but an important cause of long-term morbidity. Neuro-Behcet’s disease is more common in males and neurological symptoms typically present 3 to 6 years after other systemic manifestations [93,94]. Movement disorders including chorea and parkinsonism have been reported in neuro-Behcet’s disease and are believed to be related to anti-basal ganglia antibodies [95]. Treatment recommendations are for steroid administration in case of an acute attack, which should be continued for at least 6 months. Other immunosuppressive agents should also be administered to young patients or those who develop neurological manifestations after a short latency to prevent recurrence and progression [93].

#### Sjögren syndrome (SS)

Choreic movements are rare extraglandular symptoms of SS. Current data suggest that chorea associated with SS can be isolated or present in combination with neuropsychiatric symptoms and radiological findings [96]. Evidence shows that the presence of neurologic disease in SS is a strong indicator of disease activity and damage. In these cases, early initiation of treatment has contributed to good recovery [97].

#### Celiac disease

Celiac disease is a gluten-induced immune-mediated enteropathy. The inflammatory process is triggered by the ingestion of gluten present in wheat, barley, and rye in genetically predisposed individuals, and causes predominantly gastrointestinal symptoms [98]. Extra-intestinal manifestations are not uncommon; neurological symptoms are present in 10% of patients including ataxia, myalgias, or peripheral neuropathies. Chorea is rarely reported [99,100]. In patients in whom celiac disease is suspected, measurement of serum IgA antibodies to tissue transglutaminase is considered the first screening test. IgA antiendomysial antibody test is confirmatory. A strict gluten-free diet is the treatment of choice [98].

#### Other

Antibodies against synaptic receptors and neuronal cell surface adhesion molecules such as IgLON5, D2R, GABAaR, and Neurexin-3 alpha have been found recently to cause a wide spectrum of symptoms including encephalitis, movement disorders (including chorea), neuropsychiatric manifestations, and sleep disorders [101,102,103,104].

### 4. Endocrine and Metabolic

#### Hyperthyroidism

Hyperthyroidism, in particular Grave’s disease, can also be a rare cause of acquired chorea. Young female patients (average mid-20s) are more commonly affected [105]. Chorea in hyperthyroidism has a varied presentation in terms of onset, distribution, and severity. Additional neurological and psychiatric signs associated with thyrotoxicosis are common. Chorea most commonly develops simultaneously with or after the clinical symptoms of hyperthyroidism. Signs and symptoms of hyperthyroidism, such as tachycardia and other cardiovascular symptoms, in the presence of chorea should alert the clinician to the possibility of this diagnosis. The possible underlying mechanism is related to circulating thyroid hormones [106]. Management is centered on normalization of thyroid function with antithyroid drugs. A few patients may require anti-choreic agents such as neuroleptics and tetrabenazine [107].

#### Hypocalcemia

Hypocalcemia as cause of chorea is more commonly seen in hypoparathyroidism, either idiopathic, postoperative, or pseudo-hypoparathyroidism [108]. Other reported causes of chorea secondary to hypocalcemia include malabsorption or bisphosphonate treatment [109,110,111]. Chorea is usually generalized and patients may show other manifestations of hypocalcemia. Normalization of blood calcium levels is the main treatment. Dopamine modulating agents are sometimes required.

#### Hyper-or Hypo-natremia

Serum sodium disturbances must be considered along with the status of the extracellular volume hypovolemia, euvolemia, and hypervolemia. Neurological symptoms include confusion, neuromuscular excitability, hyperreflexia, seizures, or coma. Chorea may appear during the hyper- or hyponatremic phase or after correction of the electrolyte disturbance [112].

Chorea in association with hyponatremia has been reported in intracranial tuberculomas [113]. A rapid correction of hyponatremia can cause central pontine and extrapontine myelinolysis which can also cause movement disorders [114,115,116].

#### Hypomagnesemia

Low magnesium blood levels may result in similar neurological symptoms to hypocalcemia such as increased deep tendon reflexes and presence of Chvostek’s sign [1]. Chorea is not rare, and usually occurs in the setting of other neurological features. The main causes of low magnesium blood levels are a deficient oral intake (e.g. parenteral nutrition), diarrhea, renal disease, diuresis, acute pancreatitis, or hypercalcemia. Treatment is focused on correcting the deficiency of magnesium.

#### Uremia

Chorea has been reported in patients with uremia [117], and is more commonly seen among people of East Asian ancestry, for reasons which are unknown. It can be difficult to differentiate whether the chorea is secondary to nonketotic hyperglycemia, which is usually present, or uremia; however, the observation of hyperintense lesions involving the basal ganglia associated with marked surrounding edema on MRI T2-weighted sequences, and the relatively younger age of the patient can facilitate the diagnosis. Chorea may improve after correction of uremia however, the movement has been reported to persist after resolution of the metabolic derangement [118].

#### Non-wilsonian hepatolenticuar degeneration

Acquired hepatocerebral degeneration is a progressive disorder seen in patients with advanced liver disease and portosystemic shunts [119]. Features of hepatic disease usually precede neurological symptoms. Neurological symptoms include cognitive impairment, speech problems, movement disorders, paratonia, ataxia. Tremor, myoclonus, chorea, and athetosis can be seen [120]. Chorea is present in about 20% of patients. Patients can also develop pyramidal signs and paraparesis. This disorder usually presents in middle-aged adults. Diffuse bilateral hyperintensities and cavitations in the basal ganglia on MRI and evidence of liver failure indicate the diagnosis [121].

#### Kernicterus

Kernicterus describes a chronic encephalopathic syndrome in neonates as a result of excessively elevated bilirubin leading to movement disorders, auditory dysfunction, oculomotor abnormalities, dental enamel hypoplasia, and gastrointestinal abnormalities [122]. The aggressive treatment of perinatal hyperbilirubinemia has led to a decline in the incidence kernicterus [123]. Dystonia and athetosis are more commonly reported than chorea. Brain MRI may show hyperintense lesions in the globus pallidus in T1-weighted sequences. Supportive treatment and deep brain stimulation (DBS) may provide some improvement [124].

### 5. Nutritional

#### Vitamin B12 and B1 deficiency

Movement disorders related to vitamin deficiencies are rare. Chorea is not a typically recognized characteristic of vitamin B12 deficiency. When present, it be unilateral or generalized in distribution, and is more commonly seen in male patients [125]. However, the most prevalent symptoms of vitamin B12 deficiency include vibratory and proprioceptive impairment, cognitive impairment, dizziness, muscle cramps, ataxia, erectile dysfunction, fatigue, psychiatric symptoms, and macrocytic anemia [126]. The mechanism of chorea induced by vitamin B12 deficiency is not well understood [127]. Thiamine deficiency is also a rare cause of nutritional chorea (likely due to a similar mechanism as that seen with disorders of thiamine metabolism). Patients usually present with altered mental status, ataxia, and oculomotor abnormalities, in addition the movement disorder. Brain MRI may show hyperintensities in both thalami on FLAIR sequence [128]. Chorea usually resolves after correction of nutritional deficiency.

### 6. Multiple Sclerosis (MS) and other demyelinating diseases

Chorea has been rarely reported during the course of established MS [129]. Patients are typically young and female with brain MRI findings may show demyelinating lesions in the region of the basal ganglia, especially in the contralateral striatum [130]. In most cases the chorea improves or disappears after the MS relapse. If the movement persists, dopamine receptor blockers or depletors or low doses of carbamazepine can be used [131]. Chorea has also been rarely reported in other demyelinating disorders including acute disseminated encephalomyelitis, central pontine myelinolysis and extrapontine myelinolysis, and hypomyelination with atrophy of the basal ganglia and cerebellum. The impact of treatment in the chorea of these other demyelinating disorders is still not well understood [131].

### 7. Paraneoplastic

Chorea should be suspected as being due to a paraneoplastic origin when symptoms develop in a subacute manner, in adult patients above 50 years of age, male gender, and in the presence of other neurological and systemic symptoms such as peripheral neuropathy and weight loss [132]. Paraneoplastic chorea is associated with antibodies directed against intracellular antigens including anti-Hu, anti-Ma, anti-CRMP5/CV2, and anti-P/Q and N-type voltage-gated calcium channels, and with antibodies directed against cell surface antigens including anti-NMDAR, anti-LGI1, and anti-Caspr2 [133,134]. The most common associated cancer is small cell lung cancer and the most frequently associated antibody is CRMP5 [82]. The treatment and prognosis are highly related to the treatment of the underlying malignancy. Patients typically have a short survival [84,135].

### 8. Infectious

#### Bacterial

Some of the reported uncommon bacterial causes of chorea include typhoid fever [136], pertussis, diphtheria, Legionnaires’ disease [137], tuberculous meningitis [138,139], and mycoplasma [140]. The mechanism is suggested to be related to a cytotoxic effect of bacteria [137]. Bacterial chorea develops during the course of the infection, is associated with systemic symptoms, and typically affects young patients.

#### Viral

Viral infections causing chorea include measles [141], rubella, varicella [142], mumps, influenza A [143], ECHO virus 25 [144], herpesvirus [145], Epstein-Barr [146], citomegalovirus, Japanese encephalitis [147], tick-borne encephalitis [148], and HIV [149,150]. In general, chorea secondary to viral infections typically develops over the course of the viral infection. Clinicians should suspect a viral etiology if the chorea presents in an acute or subacute fashion, and if associated with encephalopathy or other systemic signs and symptoms of the viral infection. Whether the mechanism is cytokine-mediated or cytotoxic effects of the virus is still under discussion [143]. Chorea can be unilateral or can be generalized in distribution and is usually transitory, remitting in days to weeks after the infection.

#### Spirochetal

Syphilis is a sexually transmitted chronic multisystemic disease which can present with a variety of systemic and neurological symptoms. Only a few patients affected with syphilis have been reported to develop movement disorders, and most are associated with HIV infection [151,152]. Chorea is rare and resolves with treatment of the disease. Similar to the previously mentioned spirochete, Lyme disease can cause a variety of neurological symptoms, but movement disorders, specifically chorea, are rare [153]. Treatment of neuroborreliosis usually resolves neurological and non-neurological symptoms.

### 9. Brain Hypoxia

#### Post-pump chorea

Chorea has also been reported in children, and occasionally adults, undergoing cardiopulmonary bypass surgery [154]. This syndrome is known as “post-pump chorea” or “CHAP syndrome” (choreoathetosis, oral-facial dyskinesias, hypotonia, pseudobulbar signs). Chorea is often mild, typically develops 7–12 days after the procedure with gradual resolution of chorea after several week or months [155]. Several risk factors for post-pump chorea syndrome have been reported including patients undergoing hypothermic ischemic arrest, relative polycythemia, right ventricular outflow tract obstruction with ventricular septal defect, and very rapid cooling inducing hypothermia [155].

### 10. Other

#### Mastocytosis

Mastocytosis is a rare disorder caused by excessive production and accumulation of defective mast cells and CD34+ mast cell precursors [156]. It manifests in a variety of forms with an increased risk of anaphylaxis. Systemic mastocytosis is commonly seen in adults. Neurological manifestations commonly include headache, seizures, dizziness, and cognitive impairment. Chorea is an extremely rare manifestation. It usually affects the upper body and remits after treatment of the underlying condition [157].

#### Chorea gravidarum

Chorea gravidarum is a neuropsychiatric disorder occurring in approximately 1 per 2,000 to 3,000 pregnancies [158]. Chorea may be mild to severe with symptoms beginning in the first or early second trimester. In most cases, chorea usually resolves by the third trimester or halts within hours of delivery. Psychiatric symptoms may include personality changes, depression, tourettism, hallucinations, delirium, or cognitive impairment. Collagen vascular disorders and a history of Sydenham’s chorea are frequently associated with the disorder. Other commonly associated disorders are SLE, APS, thyrotoxicosis, drug-induced chorea, Wilson’s disease, or HD. Chorea gravidarum may recur in later pregnancies. Chorea, likely due to a similar mechanism, can be seen with the use of oral contraceptives or topical estrogen. If needed, symptomatic therapy for chorea with dopamine receptor-blocking or -depleting agents are used [159].

## Conclusions

The diagnosis of chorea can be challenging. In order to promote awareness of rare disorders that can cause chorea, we provide clues to the diagnosis, clinical and radiological characteristics of rare causes of chorea. Consideration of the age of onset and, if possible, whether the disorder is hereditary or acquired, will guide the diagnostic evaluation. The most common causes of rare disorders resulting in chorea are genetic, and these are often neurodegenerative. The field of genetic causes of choreas or other movement disorders is in constant evolution, however, it is critical to identify the acquired choreas since these are treatable conditions.
